# THE EFFECT OF ACTIVITIES OF DAILY LIVING ON FAMILY CAREGIVER SOCIAL ISOLATION

**DOI:** 10.1093/geroni/igz038.3286

**Published:** 2019-11-08

**Authors:** Tony Gallanis

**Affiliations:** Framily Health, Boston, Massachusetts, United States

## Abstract

Social isolation has been shown to associate with negative health outcomes including depression and stress. For family caregivers of older adults, the demands on the caregiver often are associated with increasing feelings of loneliness and decreased social contact. The degree to which the caregiver’s social isolation is related to the complexity of the caregiving situation remains unknown. Through a cross-sectional analysis of 526 family caregivers from the Family Caregiver Alliance client record database, an association has been established between care recipient functional decline and caregiver social isolation. Social isolation was measured through the Lubben Social Network Scale and functional decline was measured through ADL/IADL reporting. Covariates controlled for in the analysis included caregiver ethnicity, duration of caregiving, adult child status, caregiver education, care recipient income, and hours per week caregiving. Family caregivers of care recipients with higher functional decline experienced elevated odds of social isolation as compared to family caregivers of care recipients with little to no functional decline. The results from this study highlight the need for medical personnel and non-profit actors to anticipate social isolation as a risk factor for family caregivers of older adults given the care recipient is experiencing functional decline.

